# Supplementary material for: Increasing efficiency of SVMp+ for handling missing values in healthcare prediction
Source: PLOS Digit Health. 2023 Jun 29;2(6):e0000281. doi: 10.1371/journal.pdig.0000281 (PMC10309617; doi:10.1371/journal.pdig.0000281)
Supplement: Attachment — Submitted filename: PLOS l2-SVMp+.pdf [file pdig.0000281.s004.pdf]

Comments to Authors:

This study offers an efficient algorithm for handling missing values when utilizing Electronic Health Records (EHR) to develop Clinical Decision Support Systems (CDSS). The authors proposed algorithm for solving the kernel SVM-based framework (l_2-SVMp+) by adding an l_2-*regularizer* to the original formulation, converting the problem into a one-class SVM. Experiment on two different tasks validated the superiority of l_2-SVMp+ over common approaches for handling missing values by showing high efficiency and accuracy.

Introduction:
Page 2 line 21: the authors should cite the paper about LUPAPI when they mentioned it for the first time.
Page 3 line 48: SMO algorithm was mentioned for the first time without any reference about it.

Methods:
Equation (1): there is no explanation about ξ, is it the same as ζ?
ζ as the slack variables or the correcting function allow misclassification with penalty hyperparameter C to determine decision boundaries. Then, could data with privileged information be given more weight which influence the decision boundary?

Dataset:
Page 6 line 91: After removing patients who had missing values, the derived dataset contains 848 patients. But, in Table 1, there are 779 patients based on gender (male 459 and female 320). Meanwhile based on age, 938 patients in total were analyzed. Please ensure the number of datasets used for this study.

Page 8: It is better to add figure of dataset split method for MNIST+ as well since it has different size of training, validation, and test sets.

Experiments and Results:
Figure 2 should be placed in page 9 before Table 3 to make it easy to read for the reader.

Page 9 line 105: The authors stated that there was a 5% jump in AUC performance when PI was included in model training. However, the AUC performance for SVMp+ was at least 6.7% while for l_2-SVMp+ increased at least 10.22%.

Page 9 line 107: The authors mentioned that when using l_2-SVMp+, the F1 value increases to 0.872 under 50% availability and gradually grows to 0.982. But, Table 3 showed that the highest F1 score was 0.892.

Page 11: Comparison of model performance in Table 4 showed that highest AUC value achieved by the proposed method was 0.596. AUC value tells how much the model is capable of distinguishing between classes. Based on a paper by Yang, et al [1], AUC value in 0.6-0.7 range means not good. Thus, the authors may elaborate more on F1 score and training time which was significantly reduced.

Discussion:

Page 12 line 123: The authors introduce the term SHAP for the first time and need to expand the abbreviation here.

Reference:

[1] Yang J, Liu X, Ai D, Fan J, Zheng Y, Li F, et al. (2015) PET Index of Bone Glucose Metabolism (PIBGM) Classification of PET/CT Data for Fever of Unknown Origin Diagnosis. PLoS ONE 10(6):e0130173. doi:10.1371/journal.pone.0130173.
