# Supplementary material for: Increasing efficiency of SVMp+ for handling missing values in healthcare prediction
Source: PLOS Digit Health. 2023 Jun 29;2(6):e0000281. doi: 10.1371/journal.pdig.0000281 (PMC10309617; doi:10.1371/journal.pdig.0000281)
Supplement: Attachment — Submitted filename: Response to Reviewers.docx [file pdig.0000281.s005.docx]

Response to Reviewers

Dear reviewers,

Thank you for reviewing the manuscript **Increasing efficiency of SVMp+ for handling missing values in healthcare prediction** for publication in PLOS Digital Health. We sincerely appreciate the time and effort taken to review our paper and provide insightful and constructive comments. We carefully considered and addressed the comments and suggestions provided by the reviewers. We appreciate all the comments, as it helped us to redesign the experiments and improve the current proposed algorithm. Our point-by-point response to the reviewers’ comments and concerns is provided below, along with a tracked changed version of the manuscript that highlights all changes. We also produce a final version of the revised manuscript, with all line numbers included below referring to the final untracked version.

**Response to Reviewer 1:**

(1)

Comment:

*Introduction*

*Page 2 line 21: the authors should cite the paper about LUPAPI when they mentioned it for the first time.*

*Page 3 line 48: SMO algorithm was mentioned for the first time without any reference about it.*

Response

Thank you for pointing out that there are no references for LUPAPI framework and SMO algorithm when they are first mentioned in the manuscript. We added the references respectively.

(2)

Comment:

*Methods*

*Equation (1): there is no explanation about ξ, is it the same as ζ?*

*ζ as the slack variables or the correcting function allow misclassification with penalty hyperparameter C to determine decision boundaries. Then, could data with privileged information be given more weight which influence the decision boundary?*

Response

Yes. ξ here is the typo for ζ. I fixed it in the manuscript. Here, ζ means the slack variable who controls the penalty for misclassification with the hyperparameter C.

Certainly, it's possible to assign greater significance to observations that contain privileged information [1]. However, in our current research, we opted to allow the optimization algorithm to autonomously minimize the cost function in an unbiased manner, as we lack prior knowledge of the missing mechanisms. The missingness might stem from a variety of causes, such as physicians deeming the measurements from prior visits to be ordinary and not worth measuring this time or failing to document the condition or record error. As a result, we prefer not to incorporate subjective knowledge into the medical prediction issue at hand. But these are really helpful suggestions, and we will consider assigning weights to samples with PI under other suitable circumstances.

(3)

Comment:

*Dataset*

*Page 6 line 91: After removing patients who had missing values, the derived dataset contains 848 patients. But, in Table 1, there are 779 patients based on gender (male 459 and female 320). Meanwhile based on age, 938 patients in total were analyzed. Please ensure the number of datasets used for this study.*

Response

Apologies for this error - 848 is the number of the patients who were non-emergency and whose discharging department and admission ward were both labeled as “cardiology”. 779 is the number of the patients within this cohort but with no missing values in the selected features. Age distribution is calculated based on the original dataset. We updated the numbers in the patient characteristics table based on the final dataset we used (779 patients).

(4)

Comment:

*Dataset*

*Page 8: It is better to add figure of dataset split method for MNIST+ as well since it has different size of training, validation, and test sets.*

Response

Thank you for your suggestions. As your recommendations, we have incorporated figures that depict the process of data partition and gradual inclusion of the ratio of privileged information (PI) into the training dataset. The PI ratio in the training dataset was systematically increased from 0.5 to 1.0, where each subsequent ratio included the same PI information as the previous one, but with additional PI.

(5)

Comment:

*Experiments and Results:*

*Figure 2 should be placed in page 9 before Table 3 to make it easy to read for the reader.*

Response
Thank you for your suggestions. The figure and table orders were updated.

(6)

Comment:

*Experiments and Results:*

*Page 9 line 105: The authors stated that there was a 5% jump in AUC performance when PI was included in model training. However, the AUC performance for SVMp+ was at least 6.7% while for l2-SVMp+ increased at least 10.22%.*

Response

Thank you for pointing this out. The AUC performance for SVMp+ was at least 6.7% increase while for *l2-SVMp+* the increase was at least 10.2%. We just used a very rough threshold to describe the behavior of the SVM model with privileged information. In the revised manuscript, we have incorporated your statement to convey the results more accurately.

(6)

Comment:

*Experiments and Results:*

*Page 9 line 107: The authors mentioned that when using l2-SVMp+, the F1 value increases to 0.872 under 50% availability and gradually grows to 0.982. But, Table 3 showed that the highest F1 score was 0.892.*

Response

Thank you for pointing this out. The reported F1 score of 0.892 is accurate, and the value of 0.982 was a typo. the manuscript is corrected accordingly.

(7)

Comment:

*Experiments and Results:*

*Page 11: Comparison of model performance in Table 4 showed that highest AUC value achieved by the proposed method was 0.596. AUC value tells how much the model is capable of distinguishing between classes. Based on a paper by Yang, et al [1], AUC value in 0.6-0.7 range means not good. Thus, the authors may elaborate more on F1 score and training time which was significantly reduced.*

Response

Thank you for your comments and suggestions. Usually, the models with AUC and AUPRC higher than 0.7 can be considered as good ones. However, this dataset is naturally hard to build a good binary classifier [2,3,4] and we only focused on the increase in terms of AUC, AUPRC and F1 score. In the original manuscript Line 112-115, we analyzed the training time in detail.

‘*It is worth noting that the SVMp+ model is unable to provide any performance metric due to its inability to converge in training after 48 hours, indicating poor scalability in implementation. The training times for the rest of the models are mostly less than 1 second.’*

To further verify the effectiveness of the proposed method in addressing missing values, we conducted an additional experiment on the UCI heart failure dataset, as the F1 scores for the PhysioNet heart failure dataset did not exhibit significant improvement compared to the other three models. We utilized age, sex, cp, trestbps, chol, fbs, restecg, thalch, exang, oldpeak as main features and slope as privileged feature to predict the presence of heart disease in patients. We added a new subsection to the Dataset section and another subsection to the Experiments and Results section in the manuscript to present the findings of this experiment. Our results show that with this dataset, we were able to develop a classifier with an AUC of approximately 0.8 and an AUPRC of roughly 0.6. The experiment demonstrated that the l2-SVMp+ model can enhance the performance of the standard SVM and, under certain circumstances, even surpass the two conventional imputation methods, indicating its potential in handling missing values in healthcare data.

We also adapted the abstract, introduction, discussion accordingly since we added a new dataset for algorithm validation.

(8)

Comment:

*Discussion:*

*Page 12 line 123: The authors introduce the term SHAP for the first time and need to expand the abbreviation here.*

Response

Thank you for your suggestions. We added one sentence to describe what SHAP is and how it can help to interpret the model.

**Response to Reviewer 2:**

(1)

Comment:

*I would suggest the authors to include the training, testing and validation results of the metrics used for the SVM (both imputation ways), SVMp+ and their proposed L2-SVMp+ models. As this would test the actual effect of the use of the LUPAPI.*

Response

Thank you for your suggestions. We added the table regarding the model performance on testing and validation datasets for four methods handling partially privileged information. Since our study focus on the generalizability and how the testing performance is increased compared to training without privileged information, we added the full table to the supplementary files and keep the original one in the manuscript.

(2)

Comment:

*I would suggest to add some illustrations for the easy understand the transition from training weights including PI to the testing weights without the PI.*

Response

Thank you for the comments. After the training is finished, the weights for constructing decision boundaries (w) and the weights for slack functions (w*) are all fixed. w* and w optimization is interacting with each other only during the training. When we are making predictions on the test dataset, the only weights we used is the w and it is fixed. Therefore, w is the same at both training and testing stage.

[1] Lapin M, Hein M, Schiele B. Learning using privileged information: SVM+ and weighted SVM[J]. Neural Networks, 2014, 53: 95-108.

[2] Pereira, Ricardo Cardoso, Pedro Henriques Abreu, and Pedro Pereira Rodrigues. "Partial Multiple Imputation With Variational Autoencoders: Tackling Not at Randomness in Healthcare Data." *IEEE Journal of Biomedical and Health Informatics* 26.8 (2022): 4218-4227.

[3] Psychogyios, Konstantinos, et al. "Missing value imputation methods for electronic health records." *IEEE Access* 11 (2023): 21562-21574.

[4] Sheng S, Xu F, Zhang Y, et al. Charlson Comorbidity Index is correlated with all-cause readmission within six months in patients with heart failure: a retrospective cohort study in China[J]. BMC Cardiovascular Disorders, 2023, 23(1): 1-9.
